# Concomitant proton pump inhibitors and capecitabine-based chemoradiotherapy in rectal cancer: an ancillary study from the PRODIGE 23 trial

**DOI:** 10.1016/j.esmogo.2024.100119

**Published:** 2025-01-03

**Authors:** M. Bridoux, E. Aymes, T. Conroy, S. Gourgou, A. Carnot, C. Borg, M.-C. Le Deley, A. Turpin

**Affiliations:** 1 Gastroenterology Department, Groupe Hospitalier Seclin Carvin, Seclin, France; 2 Department of Medical Oncology, Lille University Hospital, Lille, France; 3 Methodology and Biostatistics Unit, Centre Oscar Lambret, Lille, France; 4 Department of Medical Oncology, Institut de Cancérologie de Lorraine, Vandoeuvre-lès-Nancy, France; 5 Université de Lorraine, Inserm, INSPIIRE, Nancy, France; 6 Biometrics Unit, Institut du Cancer Montpellier, Montpellier, France; 7 French National Platform Quality of Life and Cancer, Montpellier, France; 8 University of Montpellier, Montpellier, France; 9 Department of Medical Oncology, Centre Oscar Lambret, Lille, France; 10 INSERM, EFS BFC, UMR1098, RIGHT, University of Bourgogne Franche-Comté, Interactions Greffon-Hôte-Tumeur/Ingénierie Cellulaire et Genique, Besançon, France; 11 Department of Medical Oncology, University Hospital of Besançon, Besançon, France; 12 Clinical Investigational Center, CIC-1431, Besançon, France; 13 ITAC Platform, University of Bourgogne Franche-Comté, Besançon, France; 14 CESP, INSERM, Paris-Saclay University, Paris-Sud University, UVSQ, Villejuif, France; 15 CNRS, INSERM, University Lille, CHU Lille, Institut Pasteur de Lille, UMR9020U1277-CANTHER-Cancer Heterogeneity Plasticity and Resistance to Therapies, Lille, France

**Keywords:** capecitabine-based chemoradiotherapy, neoadjuvant chemoradiotherapy, proton pump inhibitors, overall survival, recurrence-free survival

## Abstract

**Background:**

Approximately one in five cancer patients use proton pump inhibitors (PPIs). In this work, we studied the effect of PPI co-medication during neoadjuvant chemoradiotherapy (NCRT) for locally advanced rectal cancer (LARC) based on the phase III PRODIGE 23 trial.

**Materials and methods:**

We gathered data on PPI exposure for patients from the PRODIGE 23 trial. PPI exposure was defined as PPI use during capecitabine treatment, either during CRT or as adjuvant therapy. The oncological outcomes included recurrence-free survival (RFS), overall survival (OS), cumulative incidence of metastatic recurrence, and pathological response to preoperative treatment. The association of PPI use with RFS, metastatic recurrence, and histological complete response was evaluated using univariate and multivariate Cox models.

**Results:**

We analyzed data from 332 patients [165 in the NAC group with mFOLFIRINOX, capecitabine-based (CAP50) CRT, and surgery, and 167 in the standard-of-care control arm with CAP50 CRT and surgery]. Thirty-eight patients were co-administered a PPI during capecitabine administration. After a median follow-up of 49.4 months, the 3-year RFS rates were 74.1% [95% confidence interval (CI) 68.6% to 78.8%] and 68.4% (95% CI 51.2% to 80.7%) in the non-PPI and PPI groups, respectively, with no significant differences (*P* = 0.16). The 3-year OS rates were 91.2% (95% CI 87.2% to 93.9%) and 83% (95% CI 65.7% to 92%) in the non-PPI group and PPI groups, respectively, with no significant differences (*P* = 0.38). We observed no difference in the pathological complete response rate between both groups.

**Conclusions:**

We observed no significant association between PPI exposure and survival or pathological response of patients receiving capecitabine-based CRT for LARC, supporting earlier research findings.

## Highlights

• Ancillary study of Prodige23 trial to investigate the association between PPIs and outcomes in LARC patients receiving CRT.
• A tendency for decreased survival in patients taking PPIs despite the lack of statistical significance was observed.
• PPIs did not affect the pathological response of patients.
• Due to the many drug interactions, PPIs should be prescribed with caution in patients with medical indications.

## Introduction

Proton pump inhibitors (PPIs) are the most commonly prescribed drugs worldwide and are used by ∼20% of patients with cancer.^1^^,^^2^ PPIs irreversibly inhibit H^+^/K^+^ adenosine triphosphatase pumps in the gastric parietal cells, suppressing gastric acid production. PPIs are known to be involved in numerous drug–drug interactions (DDIs) through the following mechanisms: (i) reduction of gastric acidity (decrease in the absorption of weakly basic drugs), (ii) inhibition of hepatic cytochromes (resulting in increased systemic exposure to certain drugs), and (iii) modification of the gut microbiome.^2^^,^^3^ The first well-established DDI between PPIs and cancer treatments involved tyrosine kinase inhibitors. This is because PPIs increase the gastric pH, reducing the absorption and bioavailability of weakly basic drugs. This decrease in bioavailability can sometimes lead to decreased efficacy, as seen in drugs like erlotinib, gefitinib, pazopanib, and sunitinib.^2^^,^^4^ As capecitabine has optimal absorption under acidic conditions, it has been speculated that an increase in the gastric pH may lead to reduced dissolution and absorption of capecitabine tablets.^5^ However, the impact of PPI intake on capecitabine efficacy remains controversial.

Conflicting data exist regarding the impact of PPI co-medication on locally advanced rectal cancer (LARC) treated with capecitabine-based neoadjuvant chemoradiotherapy (NCRT). In retrospective studies, Zhang et al. found improved tumor regression grades in PPI users compared with non-users.^6^ Cesca et al. found no modification in the complete pathologic response rate (ypT0N0 rate: 29.4% with PPIs versus 19.5% without; *P* = 0.13),^7^ and our group found a non-significant reduction (8.7% versus 19%; *P* = 0.36).^8^ Additionally, Zhang et al. reported a reduced recurrence rate,^6^ whereas Menon et al. found no difference in recurrence-free survival (RFS) or overall survival (OS) between PPI users and non-users.^8^^,^^9^

NCRT and surgical procedure improvements have significantly reduced the rate of local recurrences in LARC^10^; however, metastatic recurrence remains a significant problem. The PRODIGE 23 study, published in 2020, was the first to demonstrate a significant improvement in disease-free survival, metastasis-free survival, and OS with the addition of total neoadjuvant therapy.^11^ Updated results after 7 years were recently presented at the American Society of Clinical Oncology 2023.^12^

In this work, we carried out an ancillary study of the prospective PRODIGE 23 trial to investigate the association between PPI co-medication and outcomes in patients with LARC.

## Materials and methods

PRODIGE 23 was a multicenter randomized phase III clinical trial that investigated the role of neoadjuvant chemotherapy (NAC) with six cycles of modified FOLFIRINOX (mFOLFIRINOX) before capecitabine-based chemoradiotherapy (CRT), total mesorectum excision (TME), and adjuvant chemotherapy (NAC arm) versus CRT, TME, and adjuvant chemotherapy (standard-of-care, control arm) in patients with resectable T3 or T4 LARC. The study diagram is summarized in Supplementary Figure S1, available at https://doi.org/10.1016/j.esmogo.2024.100119. The data used in this study were extracted from the database of the UNICANCER-PRODIGE 23 trial (NCT01804790).^11^

All patients included in the randomized trial were expected to receive capecitabine-based (CAP50) CRT^13^; some of them could also receive capecitabine as adjuvant treatment. In this analysis, we selected all patients who received capecitabine and for whom information on concomitant medications was available. For each patient, PPI exposure was defined as PPI use concomitantly with capecitabine treatment administered either during CRT or as adjuvant therapy. The distribution of patient and disease characteristics was compared according to PPI exposure using the chi-square test or Fisher’s exact test for categorical data and Student’s *t*-test or Wilcoxon test for continuous variables.

The oncological outcomes included RFS, OS, cumulative incidence of metastatic recurrence, and pathological response to preoperative treatment. RFS was defined as the time from randomization until the first occurrence, which could be a locoregional recurrence, metastatic recurrence, or death from any cause.^14^ Oncological events were centrally reviewed, with outcome assessors masked until treatment allocation. Observations were censored on the date of the last follow-up in patients who were alive at the last follow-up. The cumulative incidence of metastatic recurrence was estimated by considering the time interval from inclusion to metastatic recurrence (possibly associated with locoregional recurrence), locoregional recurrence, second cancer, or death without prior metastatic recurrence as competing events. OS was defined as the time interval from the date of randomization to the date of death from any cause. Observations were censored on the date of the last follow-up in patients who were alive at the last follow-up. The median follow-up period was estimated from the inclusion/randomization date to the date of the last follow-up using the inverse Kaplan–Meier method (Schemper). The RFS and OS curves were estimated using the Kaplan–Meier method according to the PPI exposure groups.

After checking the proportional hazards assumption, the association between PPI exposure and RFS was evaluated using univariate and multivariate Cox models. In addition to PPI exposure, the multivariable model included factors such as randomized treatment arm, adjuvant chemotherapy treatment (FOLFOX versus capecitabine), and possible confounders identified through univariate analyses (variables associated with *P* values < 0.2). Based on literature data and the Princeps article of the PRODIGE 23 trial, we considered the following candidate factors: age; sex; World Health Organization performance status; tumor site (distance to anal verge: ≤5/5.1-10/>10 cm); extramural spread of the tumor in the perirectal region; American Cancer Society TNM (tumor–node–metastasis) stage (I/II versus III/IV) combining the following: initial T stage on magnetic resonance imaging, N stage, and M stage. The hazard ratios (HRs) were estimated using 95% confidence intervals (CIs). We also estimated the HR associated with PPI exposure for RFS according to the randomization group. The heterogeneity of the PPI effect according to the randomized arm was tested using an interaction term in the Cox model. A similar approach was used to investigate the association between PPI use and OS.

The cumulative incidence of metastatic recurrence was estimated using the method of Kalbfleisch and Prentice according to the PPI exposure group to consider competing events (occurrence of locoregional recurrence or death without prior metastatic recurrence). This estimate was completed by censoring the observations at the date of occurrence of a competing event to be consistent with the cause-specific Cox model used to determine the effect of PPI exposure on the risk of local/locoregional recurrence. We carried out a univariate cause-specific Cox proportional hazards model to assess the association between PPI exposure and metastatic recurrence. Events other than metastatic recurrence were considered censored observations.

Finally, we evaluated the association between PPI use and histological complete response using the chi-square test. In this analysis, PPI exposure was limited to patients who were administered PPI before surgery (CRT). Non-operated patients were considered to have treatment failures.

## Results

### Patient and tumor characteristics overall and according to PPI exposure

Among the 461 patients included in the PRODIGE 23 trial, 16 did not receive capecitabine. In addition, data on co-medications were missing for 113 patients, leaving us with a study population of 332 patients for the present analysis. As detailed in Supplementary Table S1 and Figure S2, available at https://doi.org/10.1016/j.esmogo.2024.100119, we checked the absence of significant differences between patients included in the current analysis and those who were not in terms of main baseline characteristics and outcomes. Of the 332 included patients, 165 were in the NAC arm treated with mFOLFIRINOX, CAP50 CRT, and surgery, while 167 patients were in the standard-of-care control arm treated with CAP50 CRT and surgery.

As shown in Table 1, 217 of the 332 patients (65.4%) were male. The median age at diagnosis was 61 years (range 54-67 years). A total of 322 (97%) patients had cT3 or cT4 tumors, and 301 (90.7%) had clinical lymph node involvement. Overall, most patients had a medical history of diabetes (75.3%) and arterial hypertension (75.3%) but no medical history of gastrointestinal disease (95.5%) or ulcers (98.5%).

**Table 1:** Patient and disease characteristics, overall and according to concomitant PPI exposure

Characteristics	No PPI exposure (*n* = 294)		PPI exposure (*n* = 38)		Total (*N* = 332)		*P* value
Age at randomization, years	*n* = 294		*n* = 38		*N* = 332		0.38
Median—(Q1-Q3) (range)	60.5	(54-67) (29-76)	62.0	(55-68) (26-74)	61	(54-67) (26-76)	
Mean—SD	59.7	9.1	60.5	10.3	59.8	9.3	
Sex							0.95
Male	192	65.3%	25	65.8%	217	65.4%	
Female	102	34.7%	13	34.2%	115	34.6%	
WHO performance status—(MD = 5)							0.82
0	231	79.4%	28	77.8%	259	79.2%	
1	60	20.6%	8	22.2%	68	20.8%	
Tumor site (distance to anal verge), cm							0.89
Low (≤5 cm)	106	36.1%	12	31.6%	118	35.5%	
Medium (5.1-10 cm)	150	51.0%	21	55.3%	171	51.5%	
High (10.1-15 cm)	38	12.9%	5	13.2%	43	13.0%	
Extramural spread of the tumor to the perirectal fat							0.13
<5 mm	124	42.2%	21	55.3%	145	43.7%	
≥5 mm	170	57.8%	17	44.7%	187	56.3%	
MRI T stage^a^—(MD = 6)							0.20^b^
T2	2	0.7%	2	5.3%	4	1.2%	
T3	236	81.9%	32	84.2%	268	82.2%	
T3a	20	6.9%	5	13.2%	25	7.7%	
T3b	108	37.5%	15	39.5%	123	37.7%	
T3c	90	31.3%	9	23.7%	99	30.4%	
T3d	18	6.3%	3	7.9%	21	6.4%	
T4	50	17.4%	4	10.5%	54	16.5%	
T4a	3	1.0%	0	—	3	0.9%	
T4b	47	16.3%	4	10.5%	51	15.6%	
cN at inclusion^a^							0.07
N0	27	9.2%	4	10.5%	31	9.3%	
N1	188	63.9%	30	78.9%	218	65.7%	
N2	79	26.9%	4	10.5%	83	25.0%	
Presence of distant metastasis at inclusion							0.39
No	291	99.0%	37	97.4%	328	98.8%	
Yes	3	1.0%	1	2.6%	4	1.2%	
Medical history of diabetes							0.01
No	79	26.9%	3	7.9%	82	24.7%	
Yes	215	73.1%	35	92.1%	250	75.3%	
Medical history of cardiovascular disease							0.06
No	284	96.6%	34	89.5%	318	95.8%	
Yes	10	3.4%	4	10.5%	14	4.2%	
Medical history of arterial hypertension							0.01
No	79	26.9%	3	7.9%	82	24.7%	
Yes	215	73.1%	35	92.1%	250	75.3%	
Medical history of cancer							0.58
No	288	98.0%	37	97.4%	325	97.9%	
Yes	6	2.0%	1	2.6%	7	2.1%	
Medical history of respiratory disease							0.052
No	289	98.3%	35	92.1%	324	97.6%	
Yes	5	1.7%	3	7.9%	8	2.4%	
Medical history of gastrointestinal disease							<0.001
No	288	98.0%	29	76.3%	317	95.5%	
Yes	6	2.0%	9	23.7%	15	4.5%	
Medical history of ulcer							0.01
No	292	99.3%	35	92.1%	327	98.5%	
Yes	2	0.7%	3	7.9%	5	1.5%	
PRODIGE 23 randomization group							0.28
Standard-of-care group	151	51.4%	16	42.1%	167	50.3%	
Neoadjuvant FOLFIRINOX chemotherapy group	143	48.6%	22	57.9%	165	49.7%	

The sums of the percentages may not equal 100% due to rounding.

MD, number of missing data; MRI, magnetic resonance imaging; PPI, proton pump inhibitors; SD, standard deviation; WHO, World Health Organization.

a After central review.

b Test applied between T2, T3a, T3b, T3c, T3d, T4a, and T4b proportions.

Overall, 92 (27.7%) patients received at least one PPI treatment either at the beginning or during treatment. Among them, 38 (22.8%) patients belonged to the control group, and 54 (32.7%) belonged to the NAC group. Supplementary Table S2, available at https://doi.org/10.1016/j.esmogo.2024.100119, provides further details on the PPI treatments administered to the study population, whether they were concomitant with capecitabine or not. Among the 92 patients who received at least one PPI treatment, 38 (11.4%) received PPIs concomitantly with capecitabine. The most commonly prescribed PPI was esomeprazole (42.1%) (Supplementary Table S3, available at https://doi.org/10.1016/j.esmogo.2024.100119). Among the 38 patients with concomitant PPI exposure, 30 (78.9%) had a medical indication for PPI administration (gastroesophageal reflux, ulcer, or prophylaxis with nonsteroidal anti-inflammatory drug treatment in at-risk patients).

No statistically significant difference was found in the baseline characteristics between patients with concomitant PPI exposure (*n* = 38) and the others (*n* = 294) (Table 1), except for their medical history. Patients exposed to PPIs had a higher proportion of diabetes (92% versus 73%, *P* = 0.01), arterial hypertension (92% versus 73%, *P* = 0.01), gastrointestinal disease (24% versus 2%, *P* < 0.001), and ulcer (7.9% versus 0.7%, *P* = 0.01). Patients exposed to PPIs were significantly more likely to have received at least one anti-hypertensive or antibiotic treatment during the study (60.5% versus 29.3%, *P* < 0.001 and 36.8% versus 21.4%, *P* = 0.03, respectively) but not oral antidiabetic medication (8.8% versus 7.9%, *P* = 1.0) (Supplementary Table S4, available at https://doi.org/10.1016/j.esmogo.2024.100119).

Concerning the PRODIGE 23 trial, the proportion of patients exposed to PPI did not differ significantly between the randomized groups (*P* = 0.28). We also observed no significant differences in CRT, surgery, pathological findings, or adjuvant chemotherapy between PPI users and non-users, except for a higher proportion of post-operative morbidities in the PPI group (48.6% versus 24.6%, *P* = 0.002) (Supplementary Table S5, available at https://doi.org/10.1016/j.esmogo.2024.100119).

### Survival outcomes

#### RFS

**Figure 1:**
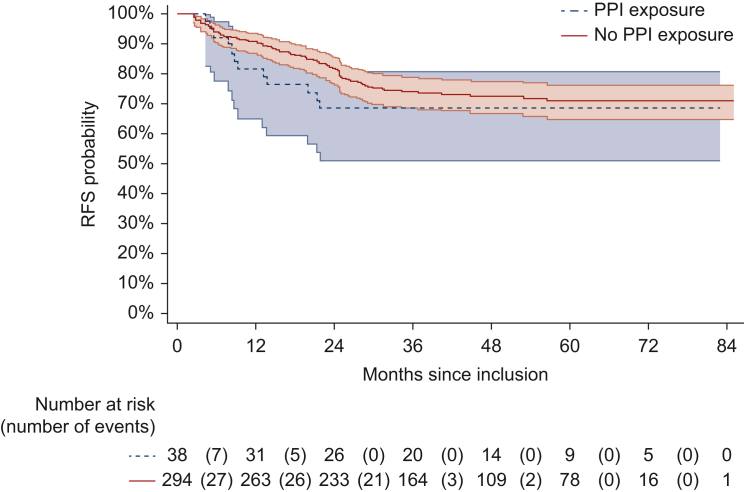
**Kaplan–Meier estimate of the recurrence-free survival curve according to PPI exposure.** PPI, proton pump inhibitor; RFS, recurrence-free survival.

After a median follow-up of 49.4 months, the 3-year RFS rates were 74.1% (95% CI 68.6% to 78.8%) in the non-PPI group and 68.4% (95% CI 51.2% to 80.7%) in the PPI group (Figure 1 and Table 2).

**Table 2:** Oncological outcomes according to concomitant PPI exposure

Oncological outcomes	No PPI exposure (*n* = 294)	PPI exposure (*n* = 38)	Overall (*N* = 332)
Recurrence-free survival (RFS)			
Number of events	**79**	**12**	**91**
Type of event			
Relapse/progression	**70**	**10**	**80**
Locoregional recurrence	13	0	13
Metastatic recurrence	54	10	64
Combined (locoregional and meta)	2	0	2
Death from disease progression with no date of disease progression^a^	1	0	1
Death without prior recurrence	**9**	**2**	**11**
Toxicity	2	0	2
Intercurrent disease	2	1	3
Death related to rectal cancer with no prior relapse or progression	5	1	6
3-year RFS estimate (95% CI)	74.1% (68.6-78.8)	68.4% (51.2-80.7)	73.5% (68.3-78.0)
Overall survival (OS)			
Number of deaths	**43**	**6**	**49**
Cause of death			
Cancer-related/progression	36	5	41
Toxicity	2	0	2
Intercurrent disease	2	1	3
Other (after relapse)	3	0	3
3-year OS estimate (95% CI)	91.2% (87.2-93.9)	83.0% (65.7-92.0)	90.2% (86.4-93.0)
Cumulative incidence of metastatic relapse/progression			
Events	**56**	**10**	**66**
Metastatic recurrence	54	10	64
Locoregional and metastatic recurrence	2	0	2
Competing event	**28**	**4**	**32**
Locoregional recurrence	13	0	13
Second cancer	6	2	8
Death	9	2	11
3-year cumulative incidence, considering other events as competing events (95% CI)	18.6% (14.3-23.3)	26.3% (13.7-40.8)	19.5% (15.3-24.0)
Pathological response (MD = 2)			
Complete pathological response (ypT0N0)	237 (80.9%)	29 (78.4%)	266
No complete response	56 (19.1%)	8 (21.6%)	64

CI, confidence interval; MD, number of missing data; PPI, proton pump inhibitors.

a For one patient, a death occurring 7.2 months after randomization was reported as related to disease progression, although no date of relapse or progression was reported. We have no details about this event. The date of event considered for the analysis is the date of death.

As detailed in Table 3, despite a trend for a worse outcome, concomitant PPI exposure was not significantly associated with RFS as evidenced by the univariate (HR 1.27, 95% CI 0.69-2.33, *P* = 0.45) or multivariable analyses (HR 1.56, 95% CI 0.84-2.88, *P* = 0.16) after adjusting for sex, randomization group, adjuvant treatment regimen, and tumor site. The results appeared relatively homogeneous across the two randomized groups (NAC and control arms), with no significant interaction between PPI exposure and the treatment arm (*P* = 0.71).

**Table 3:** Factors associated with the risk of relapse or death (recurrence-free survival)

Factors	Nb events/*N*	Crude HR	95% CI	*P* value	Adjusted HR^a^	95% CI	*P* value
PPI exposure				0.45			0.16
No	79/294	1			1		
Yes	12/38	1.27	(0.69-2.33)		1.56	(0.84-2.88)	
Age at randomization				0.41			
HR/1 year	91/332	1.01	(0.99-1.03)				
Sex				0.08			0.43
Male	67/217	1			1		
Female	24/115	0.66	(0.41-1.05)		0.82	(0.51-1.33)	
Randomization group				0.002			0.001
Standard-of-care	58/167	1			1		
Neoadjuvant FOLFIRINOX chemotherapy	33/165	0.51	(0.33-0.77)		0.46	(0.30-0.72)	
Adjuvant regimen				<0.001			<0.001
No adjuvant treatment	38/84	3.08	(2.0-4.75)		3.06	(1.98-4.74)	
FOLFOX6	45/215	1			1		
Capecitabine	8/33	1.16	(0.55-2.47)		1.15	(0.54-2.47)	
WHO performance status*—*(MD = 5)				0.18			b
0	76/259	1					
1	14/68	0.68	(0.38-1.19)				
Tumor site (distance to anal verge)				0.01			0.01
Low (≤5 cm)	44/118	1			1		
Medium (5.1-10 cm)	36/171	0.51	(0.33-0.80)		0.50	(0.32-0.79)	
High (10.1-15 cm)	11/43	0.67	(0.35-1.30)		0.63	(0.32-1.23)	
Extramural spread of the tumor to the perirectal fat				0.07			c
<5 mm	33/145	1					
≥5 mm	58/187	1.49	(0.97-2.28)				
Initial TNM stage				0.45			
I/II	7/31	1					
III/IV	84/301	1.34	(0.62-2.90)				

CI, confidence interval; HR, hazard ratio; MD, number of patients with missing data; PPI, proton pump inhibitor; TNM, tumor–node–metastasis; WHO, World Health Organization.

a The HRs were estimated using the multivariable model and included the following variables: PPI exposure, sex, randomization group, adjuvant regimen, and tumor site (distance to anal verge).

b The variable ‘WHO performance status’ was not included in the multivariable model because it was significantly associated with sex (*P* = 0.005) and tumor site (*P* = 0.01).

c The variable ‘extramural spread of the tumor to the perirectal fat’ was not included in the multivariable model because it was significantly associated with the tumor site (*P* = 0.04).

#### OS

**Figure 2:**
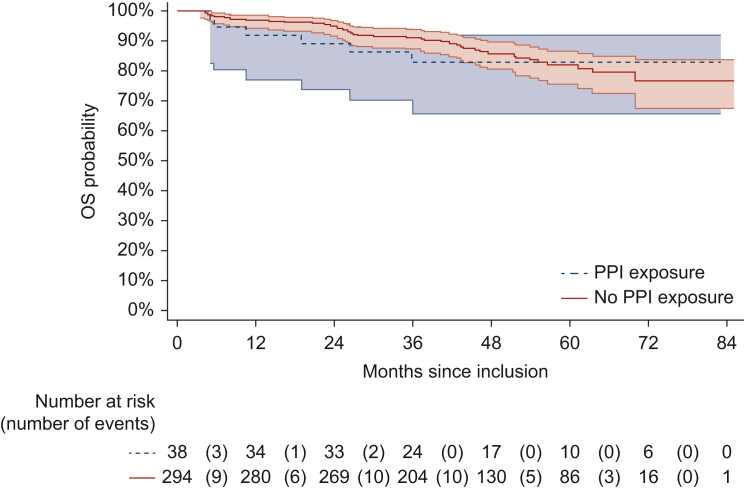
**Kaplan–Meier estimate of the overall survival curve according to PPI exposure.** OS, overall survival; PPI, proton pump inhibitor.

The 3-year OS rates were 91.2% (95% CI 87.2% to 93.9%) in the non-PPI group and 83% (95% CI 65.7% to 92%) in the PPI group (Figure 2 and Table 2).

As detailed in Supplementary Table S6, available at https://doi.org/10.1016/j.esmogo.2024.100119, despite a trend for a worse outcome, PPI exposure was not significantly associated with OS, as shown in the univariate (HR 1.12, 95% CI 0.48-2.64, *P* = 0.79) or multivariable analyses (HR 1.48, 95% CI 0.62-3.55, *P* = 0.38). The results appeared relatively homogeneous across the two randomized groups, with no significant interactions between PPI exposure and the treatment arm (*P* = 0.72).

#### Metastatic recurrence

**Figure 3:**
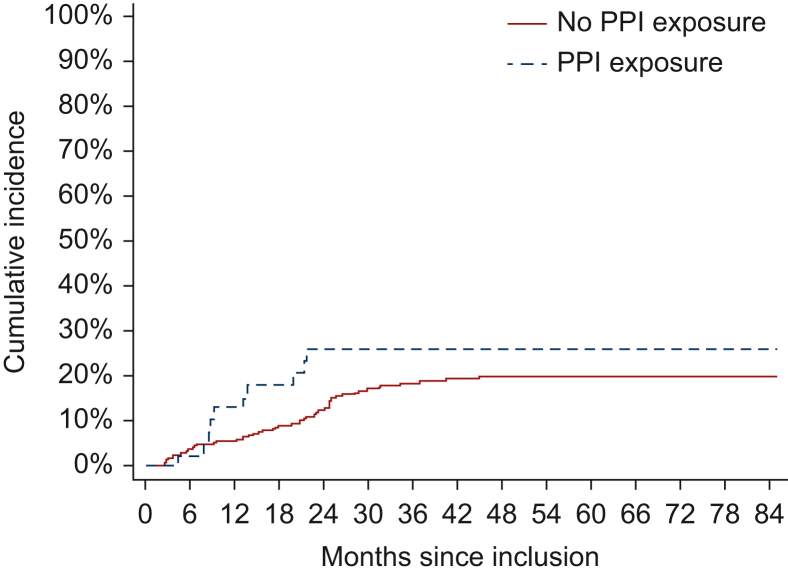
**Kalbfleisch and Prentice estimate of the cumulative incidence of metastatic recurrence according to concomitant PPI exposure.** PPI, proton pump inhibitor.

Overall, 66 patients presented with metastatic recurrence (56 in the non-PPI group and 10 in the PPI group), and 32 presented with a competing event (28 in the non-PPI group and 4 in the PPI group). The univariate cause-specific Cox model estimated a non-significant association between PPI exposure and metastatic recurrence (cs-HR 1.52, 95% CI 0.78-2.99, *P* = 0.22) (Table 2, Figure 3).

#### Pathological features

We observed no difference in the pathological complete response rate between the two groups: 8/37 patients (21.6%) who were exposed to PPIs during CRT and 56/295 patients (19.1%) who were not exposed to PPIs during CRT (*P* = 0.72).

## Discussion

Our ancillary study of the PRODIGE 23 trial showed a tendency for decreased survival (RFS and OS) in patients taking PPIs despite the lack of statistical significance. Notably, pathological complete response rates were similar between PPI and non-PPI users, an indicator of neoadjuvant treatment efficacy. Our results are consistent with those of other studies, finding no association between PPI use and pathological response,^7^, ^8^, ^9^ or any change in RFS or OS.^8^^,^^9^ A prospective study based on a clinical trial with several hundred patients found that >27% of patients reported taking at least one PPI, consistent with the literature (20%-25% of patients).^7^^,^^8^ Oncological treatment appeared similar in patients with concomitant exposure to PPIs, limiting the risk of guarantee-time bias.

We hypothesized that PPIs would reduce the absorption and efficacy of capecitabine-based chemotherapy. Several retrospective studies on colorectal cancer have shown poor survival when PPIs were combined with capecitabine.^2^^,^^15^ A retrospective study by Wong et al. on adjuvant chemotherapy for early colorectal cancer (CRC) observed a reduced 3-year RFS in patients receiving CAPOX, but not FOLFOX (HR 0.51, 95% CI 0.25-1.06).^16^ However, this potential interaction is disputed by newer pharmacokinetic and *in vitro* studies demonstrating no biological mechanism for such interaction between PPIs and capecitabine.^15^^,^^17^

The recent systematic review and meta-analysis conducted by Lin et al. involved 8188 patients with CRC (PPI = 1789; non-PPI = 6329) receiving either capecitabine-based or fluorouracil-based regimens.^18^ They found similar OS and progression-free survival (PFS) between PPI users and non-users in patients taking capecitabine-based regimens, except for early-stage cancer patients taking capecitabine monotherapy where PPI use was associated with a significantly higher disease progression rate (HR 1.96, 95% CI 1.21-3.16, I^2^ = 0%). Moreover, both groups had comparable all-cause mortality.

Although an interaction between PPIs and capecitabine is doubtful, recent data suggest interactions between PPIs and immune checkpoint inhibitors (ICIs).^2^^,^^3^ PPIs have been shown to alter the gut microbiome and reduce the efficacy of ICIs. One of the latest meta-analyses on this topic, published in 2023, included 41 studies with 20 042 patients.^19^ It reported shorter OS (HR 1.37, 95% CI 1.23-1.52) and PFS (HR 1.28, 95% CI 1.15-1.42) in cancer patients receiving PPIs than those who did not.

However, this study has some limitations. Firstly, 113 patients were excluded from the analysis owing to missing data on co-medications. In addition, PPI use may have been underreported, as drugs are available over the counter in drug stores. Given the multimodal nature of the treatment in this study, the potential interaction between PPIs and capecitabine only pertains to a specific portion of the regimen. Since no interaction is expected with radiotherapy or surgery, identifying a definitive interaction between PPIs and capecitabine may be more challenging. We also acknowledge the limited power of the comparison due to the sample size of the PRODIGE 23 trial and the relatively small proportion of patients receiving concomitant PPIs. We recognize that our results do not allow us to rule out a deleterious effect of PPI co-administration. Lastly, as we re-analyzed the data in the main publication,^11^ follow-up was still limited for the efficacy endpoint analysis.

In the ancillary analysis of the TRIO-013/LOGiC trial, a significant adverse relationship was observed between PPI co-administration with capecitabine and survival outcomes.^4^ This study is much better powered than ours, as 273 of the 545 recruited patients were allocated to the capecitabine group and 119 of these patients received concomitant PPI. However, the trial was carried out in a very different setting (HER2-overexpressed gastric cancer patients), associated with a better prognosis, more gastrointestinal symptoms, and consequently a longer exposure to PPIs.

In a future trial, incorporating a pharmacokinetic evaluation may provide useful functional assessment of capecitabine absorption, in the setting of PPI and co-administration of other drugs including other acid suppressants.

In conclusion, in this ancillary analysis of the PRODIGE 23 trial, we observed no significant difference in survival or pathological response in patients taking PPIs during capecitabine-based CRT for LARC. However, PPIs are not harmless, we must not forget their adverse effects, which can potentiate those of oncological treatments, as well as the many drug interactions in which they may be involved. Therefore, PPIs should be prescribed with caution in patients with medical indications. Meta-analysis of the interaction should be conducted, and an alternative to co-administration in the adjuvant setting should be considered while larger better powered studies are needed.
