# Supplementary material for: Concomitant proton pump inhibitors and capecitabine-based chemoradiotherapy in rectal cancer: an ancillary study from the PRODIGE 23 trial
Source: ESMO Gastrointest Oncol. 2025 Jan 3;7:100119. doi: 10.1016/j.esmogo.2024.100119 (PMC12836692; doi:10.1016/j.esmogo.2024.100119)
Supplement: Supplementary Data [file mmc1.docx]

# Supplementary Tables

Supplementary Table 1. Comparison of patient characteristics and outcomes between patients selected for the current analysis or not but within the PRODIGE 23 trial population.

| **Characteristics** | **Not selected**  **N=129** | | **Selected**  **N=332** | | **Total**  **N=461** | | **p-value** |
| --- | --- | --- | --- | --- | --- | --- | --- |
| **Sex** |  |  |  |  |  |  | p=0.46 |
| Male | 89 | 69.0% | 217 | 65.4% | 306 | 66.4% |  |
| Female | 40 | 31.0% | 115 | 34.6% | 155 | 33.6% |  |
| **Age at randomization, years** |  |  |  |  |  |  | p=0.34 |
| Median (Q1–Q3) (Range) | 61 | (54;66) (32;77) | 61 | (54;67  (26;76) | 61 | (54;66) (26;77) |  |
| Mean - SD | 58.7 | 9.6 | 59.8 | 9.3 | 59.5 | 9.4 |  |
| **WHO performance status (N=455, DM=6)** |  |  |  |  |  |  |  |
| 0 | 101 | 78.9% | 259 | 79.2% | 360 | 79.1% | p=0.94 |
| 1 | 27 | 21.1% | 68 | 20.8% | 95 | 20.9% |  |
| **Distance to anal verge, cm** |  |  |  |  |  |  | p=0.63 |
| Low (≤5 cm) | 52 | 40.3% | 118 | 35.5% | 170 | 36.9% |  |
| Medium (5.1–10 cm) | 61 | 47.3% | 171 | 51.5% | 232 | 50.3% |  |
| High (10.1–15cm) | 16 | 12.4% | 43 | 13.0% | 59 | 12.8% |  |
| **Extramural spread of the tumor to the perirectal fat** |  |  |  |  |  |  | p=0.96 |
| <5 mm | 56 | 43.4% | 145 | 43.7% | 201 | 43.6% |  |
| ≥5 mm | 73 | 56.6% | 187 | 56.3% | 260 | 56.4% |  |
| **MRI T stage (N=450, DM=11)** |  |  |  |  |  |  | p=0.74 |
| T2 | 1 | 0.8% | 4 | 1.2% | 5 | 1.1% |  |
| T3a | 9 | 7.3% | 25 | 7.7% | 34 | 7.6% |  |
| T3b | 46 | 37.1% | 123 | 37.7% | 169 | 37.6% |  |
| T3c | 38 | 30.6% | 99 | 30.4% | 137 | 30.4% |  |
| T3d | 9 | 7.3% | 21 | 6.4% | 30 | 6.7% |  |
| T4a | 4 | 3.2% | 3 | 0.9% | 7 | 1.6% |  |
| T4b | 17 | 13.7% | 51 | 15.6% | 68 | 15.1% |  |
| **cN at inclusion** |  |  |  |  |  |  | p=0.69 |
| N0 | 15 | 11.6% | 31 | 9.3% | 46 | 10.0% |  |
| N1 | 85 | 65.9% | 218 | 65.7% | 303 | 65.7% |  |
| N2 | 29 | 22.5% | 83 | 25.0% | 112 | 24.3% |  |
| **Presence of distant metastasis at inclusion** |  |  |  |  |  |  | p=0.023 |
| No | 123 | 95.3% | 328 | 98.8% | 451 | 97.8% |  |
| Yes | 6 | 4.7% | 4 | 1.2% | 10 | 2.2% |  |
| **Medical history of diabetes** |  |  |  |  |  |  |  |
| No | 39 | 30.2% | 82 | 24.7% | 121 | 26.2% |  |
| Yes | 90 | 69.8% | 250 | 75.3% | 340 | 73.8% |  |
| **Medical history of cardiovascular disease** |  |  |  |  |  |  | p=0.58 |
| No | 125 | 96.9% | 318 | 95.8% | 443 | 96.1% |  |
| Yes | 4 | 3.1% | 14 | 4.2% | 18 | 3.9% |  |
| **Medical history of arterial hypertension** |  |  |  |  |  |  | p=0.23 |
| No | 39 | 30.2% | 82 | 24.7% | 121 | 26.2% |  |
| Yes | 90 | 69.8% | 250 | 75.3% | 340 | 73.8% |  |
| **Medical history of cancer** |  |  |  |  |  |  | p=0.10 |
| No | 129 | 100.0% | 325 | 97.9% | 454 | 98.5% |  |
| Yes | 0 | 0.0% | 7 | 2.1% | 7 | 1.5% |  |
| **Medical history of respiratory disease** |  |  |  |  |  |  | p=0.26 |
| No | 128 | 99.2% | 324 | 97.6% | 452 | 98.0% |  |
| Yes | 1 | 0.8% | 8 | 2.4% | 9 | 2.0% |  |
| **Medical history of gastrointestinal disease** |  |  |  |  |  |  | p=0.05 |
| No | 128 | 99.2% | 317 | 95.5% | 445 | 96.5% |  |
| Yes | 1 | 0.8% | 15 | 4.5% | 16 | 3.5% |  |
| **Medical history of ulcer** |  |  |  |  |  |  | p=1.00 |
| No | 128 | 99.2% | 327 | 98.5% | 455 | 98.7% |  |
| Yes | 1 | 0.8% | 5 | 1.5% | 6 | 1.3% |  |
| **Arm** |  |  |  |  |  |  | p=0.78 |
| Standard-of-care group | 63 | 48.8% | 167 | 50.3% | 230 | 49.9% |  |
| Neoadjuvant chemotherapy group | 66 | 51.2% | 165 | 49.7% | 231 | 50.1% |  |
| **Recurrence-free survival** |  |  |  |  |  |  | p=0.49 |
| Hazard Ratio | 1 (ref) |  | 0.87 | (0.59–1.29) |  |  |  |

Supplementary Table 2. Description of the PPI treatments reported in the study population, concomitant with capecitabine or not (92 patients).

| **Characteristics** | **Total**  **(N=92)** | |
| --- | --- | --- |
| **PPI category***, multiple exposure possible* |  |  |
| At least one lansoprazole treatment | 9 | 9.8% |
| At least one omeprazole treatment | 20 | 21.7% |
| At least one rabeprazole treatment | 2 | 2.2% |
| At least one esomeprazole treatment | 48 | 52.2% |
| At least one pantoprazole treatment | 22 | 23.9% |
| **Number of PPI treatments sequences** |  |  |
| 1 | 79 | 85.9% |
| 2 | 12 | 13.0% |
| 3 | 1 | 1.1% |
| **Main reported reason for PPI treatment** |  |  |
| Gastro-esophagal reflux | 38 | 41.3% |
| Nausea/vomiting | 6 | 6.5% |
| Ulcer | 11 | 12.0% |
| Pain | 9 | 9.8% |
| Prophylaxis | 18 | 19.6% |
| Unspecified | 10 | 10.9% |
| **Medical indication for PPI treatment*** |  |  |
| No | 35 | 38.0% |
| Yes | 57 | 62.0% |
| **PPI use at baseline** |  |  |
| No | 74 | 80.4% |
| Yes | 18 | 19.6% |
| **PPI use concomitant to chemoradiotherapy** |  |  |
| No | 55 | 59.8% |
| Yes | 37 | 40.2% |
| **PPI use concomitant to adjuvant capecitabine chemotherapy** |  |  |
| No | 88 | 95.7% |
| Yes | 4 | 4.3% |
| **PPI use concomitant to chemoradiotherapy or adjuvant capecitabine** |  |  |
| No | 54 | 58.7% |
| Yes | 38 | 41.3% |
| **PPI treatment duration in weeks** | N=76 |  |
| Median (Q1–Q3) (Range) | 11.1 | (4.1–29.2) (0.1;1389.7) |
| Mean - SD | 39.3 | 160.9 |

**Medical indication: gastro-esophagal reflux, ulcer treatment indication, or the association of NSAID therapy in patients at risk (> 65 years old or history of ulcer or anticoagulant or antiplatelet medication)*

*PPI (proton pump inhibitor) use during the different periods of treatment are not mutually exclusive.*

Supplementary Table 3. Description of PPI use concomitant to capecitabine treatment (38 patients).

| **Characteristics** | **Total** | |
| --- | --- | --- |
| **PPI category** |  |  |
| lansoprazole | 7 | 18.4% |
| omeprazole | 7 | 18.4% |
| rabeprazole | 2 | 5.3% |
| esomeprazole | 16 | 42.1% |
| pantoprazole | 6 | 15.8% |
| **Reported reason for PPI treatment** |  |  |
| Gastro-esophagal reflux | 22 | 57.9% |
| Nausea/vomiting | 2 | 5.3% |
| Ulcer | 2 | 5.3% |
| Pain | 2 | 5.3% |
| Prophylaxis | 7 | 18.4% |
| Unspecified | 3 | 7.9% |
| **Medical indication for a PPI treatment*** |  |  |
| No | 8 | 21.1% |
| Yes | 30 | 78.9% |
| **PPI use at baseline and concomitant to Capecitabine treatment** |  |  |
| No | 20 | 52.6% |
| Yes | 18 | 47.4% |
| **PPI use concomitant to chemoradiotherapy** |  |  |
| No | 1 | 2.6% |
| Yes | 37 | 97.4% |
| **PPI use concomitant to adjuvant capecitabine chemotherapy** |  |  |
| No | 34 | 89.5% |
| Yes | 4 | 10.5% |
| **PPI treatment duration concomitant to capecitabine (weeks)** | N=38 |  |
| Median – (Q1–Q3) (Range) | 4.8 | (2.6 - 5.3)  (0.4;29.3) |
| Mean - SD | 5.7 | 6.2 |

**Medical indication: gastroesophageal reflux, ulcer treatment indication, or the association of NSAID therapy in patients at risk (> 65 years old or with a history of ulcer or anticoagulant or antiplatelet medication).*

*PPI (proton pump inhibitor) use during the different periods of treatment are not mutually exclusive.*

Supplementary Table 4. Co-medications according to concomitant PPI exposure.

| **Characteristics** | **No PPI exposure (N=294)** | | **PPI exposure**  **(N=38)** | | **P-value** |
| --- | --- | --- | --- | --- | --- |
| **At least one anti-hypertensive medication** |  |  |  |  | <0.001 |
| No | 208 | 70.7% | 15 | 39.5% |  |
| Yes | 86 | 29.3% | 23 | 60.5% |  |
| **At least one anti-arrhythmic medication** |  |  |  |  | 0.22 |
| No | 272 | 92.5% | 33 | 86.8% |  |
| Yes | 22 | 7.5% | 5 | 13.2% |  |
| **At least one anticoagulant medication** |  |  |  |  | 1.0 |
| No | 287 | 97.6% | 37 | 97.4% |  |
| Yes | 7 | 2.4% | 1 | 2.6% |  |
| **At least one antiplatelet medication** |  |  |  |  | 0.20 |
| No | 273 | 92.9% | 33 | 86.8% |  |
| Yes | 21 | 7.1% | 5 | 13.2% |  |
| **At least one oral anti-diabetes medication** |  |  |  |  | 1.0 |
| No | 268 | 91.2% | 35 | 92.1% |  |
| Yes | 26 | 8.8% | 3 | 7.9% |  |
| **At least one antibiotic medication** |  |  |  |  | 0.03 |
| No | 231 | 78.6% | 24 | 63.2% |  |
| Yes | 63 | 21.4% | 14 | 36.8% |  |

*PPI: proton pump inhibitor*

Supplementary Table 5. Anti-cancer treatment exposure and pathology findings according to concomitant PPI exposure.

| **Characteristics** | **No PPI exposure (N=294)** | | | | **PPI exposure**  **(N=38)** | | | **P-value** |
| --- | --- | --- | --- | --- | --- | --- | --- | --- |
| **PRODIGE 23 randomization group** |  |  | | |  | |  | 0.28 |
| Standard-of-care group | 151 | 51.4% | | | 16 | | 42.1% |  |
| Neoadjuvant FOLFIRINOX chemotherapy group | 143 | 48.6% | | | 22 | | 57.9% |  |
| **Duration of radiotherapy, weeks** *– (MD=1)* | N=294 |  | | | N=38 | |  | 0.82 |
| Median (Q1–Q3) (Range) | 5.3 | (5.1–5.4)  (4.4;7.6) | | | 5.3 | | (5.1–5.4)  (4.7;5.9) |  |
| Mean - SD | 5.3 | 0.4 | | | 5.2 | | 0.3 |  |
| **Interruption of radiotherapy** |  |  | | |  | |  | 0.52 |
| No | 71 | 24.1% | | | 11 | | 28.9% |  |
| Yes | 223 | 75.9% | | | 27 | | 71.1% |  |
| **Duration of Capecitabine, weeks** – *(MD=1)* | N=293 |  | | | N=38 | |  | 0.52 |
| Median (Q1–Q3) (Range) | 5.0 | (4.7–5.3)  (1.4;7.1) | | | 5.0 | | (4.7–5.3)  (4.1;5.7) |  |
| Mean - SD | 5.0 | 0.6 | | | 5.0 | | 0.3 |  |
| **At least one modified administration (for at least one week) of Capecitabine** |  |  | | |  | |  | 0.80 |
| No | 125 | 42.5% | | | 17 | | 44.7% |  |
| Yes | 169 | 57.5% | | | 21 | | 55.3% |  |
| **At least one delayed cycle (for at least one week) of Capecitabine** |  |  | | |  | |  | 1 |
| No | 272 | 92.5% | | | 35 | | 92.1% |  |
| Yes | 22 | 7.5% | | | 3 | | 7.9% |  |
| **Treated with adjuvant chemotherapy** |  | | |  |  | |  | 0.52 |
| No | 76 | | | 25.9% | 8 | | 21.1% |  |
| Yes | 218 | | | 74.1% | 30 | | 78.9% |  |
| **Adjuvant regimen^1^** |  | | |  |  | |  | 1 |
| FOLFOX6 | 189 | | | 86.7% | 26 | | 86.7% |  |
| Capecitabine | 29 | | | 13.3% | 4 | | 13.3% |  |
| **Number of adjuvant Capecitabine cycles received** |  | | |  |  | |  | - |
| 1 | 2 | | | 6.9% | 0 | | - |  |
| 2 | 3 | | | 10.3% | 0 | | - |  |
| 3 | 1 | | | 3.4% | 0 | | - |  |
| 4 | 10 | | | 34.5% | 1 | | 25.0% |  |
| 6 | 2 | | | 6.9% | 0 | | - |  |
| 8 | 11 | | | 37.9% | 3 | | 75.0% |  |
| **Number of adjuvant FOLFOX6 cycles received** |  | | |  |  | |  | - |
| 1 | 4 | | | 2.1% | 0 | | - |  |
| 2 | 1 | | | 0.5% | 1 | | 3.8% |  |
| 3 | 2 | | | 1.1% | 0 | | - |  |
| 4 | 7 | | | 3.7% | 1 | | 3.8% |  |
| 5 | 6 | | | 3.2% | 0 | | - |  |
| 6 | 84 | | | 44.2% | 16 | | 61.5% |  |
| 7 | 4 | | | 2.1% | 1 | | 3.8% |  |
| 8 | 2 | | | 1.1% | 0 | | - |  |
| 10 | 1 | | | 0.5% | 0 | | - |  |
| 11 | 6 | | | 3.2% | 1 | | 3.8% |  |
| 12 | 73 | | | 38.4% | 6 | | 23.1% |  |
|  |  | | |  |  | |  |  |
| **Surgery²** |  | |  | |  |  | | 1 |
| No | 11 | | 3.7% | | 1 | 2.6% | |  |
| Yes | 283 | | 96.3% | | 37 | 97.4% | |  |
| **Primary tumor resection²** |  | |  | |  |  | | 1 |
| No | 13 | | 4.4% | | 1 | 2.6% | |  |
| Yes | 281 | | 95.6% | | 37 | 97.4% | |  |
| **Interval between the end of CRT and surgery, days** | N=283 | |  | | N=37 |  | | 0.81 |
| Median (Q1–Q3) (Range) | 55.0 | | (48–61)  (34;106) | | 53.0 | (48–59)  (39;81) | |  |
| Mean - SD | 55.1 | | 11.1 | | 54.4 | 10.3 | |  |
| **Resection limits** *– (MD=1)* |  | |  | |  |  | | 0.68 |
| R0 | 269 | | 96.1% | | 35 | 94.6% | |  |
| R1 | 10 | | 3.6% | | 2 | 5.4% | |  |
| R2 | 1 | | 0.4% | | 0 | - | |  |
| **Tumor regression (modified Dworak)** *– (MD=44)* |  | |  | |  |  | | 0.33 |
| Grade 1 (muscularis propria) | 99 | | 41.8% | | 11 | 29.7% | |  |
| Grade 2 (intramesorectal) | 105 | | 44.3% | | 21 | 56.8% | |  |
| Grade 3 (mesorectal) | 33 | | 13.9% | | 5 | 13.5% | |  |
| **Post-operative morbidity** *– (MD=2)* |  | |  | |  |  | | 0.002 |
| No | 212 | | 75.4% | | 19 | 51.4% | |  |
| Yes | 69 | | 24.6% | | 18 | 48.6% | |  |
| **Post-operative mortality (≤30 days)** |  | |  | |  |  | | 0.39 |
| No | 280 | | 98.9% | | 36 | 97.3% | |  |
| Yes | 3 | | 1.1% | | 1 | 2.7% | |  |
| **Post-operative mortality (≤60 days)** |  | |  | |  |  | | 0.46 |
| No | 279 | | 98.6% | | 36 | 97.3% | |  |
| Yes | 4 | | 1.4% | | 1 | 2.7% | |  |

*Abbreviations: PPI, proton pump inhibitor; CRT, chemoradiotherapy treatment.*

*^1^One patient received both adjuvant chemotherapy (FOLFIRINOX and capecitabine) and was classified in the capecitabine adjuvant regimen group.*

*²There are two patients who underwent surgery but without resection.*

Supplementary Table 6. Factors associated with death.

| **Factors** | **Nb events/ N** | **Crude HR** | **95% CI** | **p-value** | **Adjusted HR^1^** | **95% CI** | **p-value** |
| --- | --- | --- | --- | --- | --- | --- | --- |
| **PPI exposure** |  |  |  | 0.79 |  |  | 0.38 |
| No | 43/294 | 1 |  |  | 1 |  |  |
| Yes | 6/38 | 1.12 | (0.48–2.64) |  | 1.48 | (0.62–3.55) |  |
| **Age at randomization** |  |  |  | 0.99 |  |  |  |
| HR/1 year | 49/332 | 1.00 | (0.97–1.03) |  |  |  |  |
| **Sex** |  |  |  | 0.017 |  |  | 0.14 |
| Male | 40/217 | 1 |  |  | 1 |  |  |
| Female | 9/115 | 0.42 | (0.20–0.86) |  | 0.57 | (0.27–1.19) |  |
| **Randomization group** |  |  |  | 0.002 |  |  | 0.002 |
| Standard-of-care | 35/167 | 1 |  |  | 1 |  |  |
| Neoadjuvant FOLFIRINOX chemotherapy | 14/165 | 0.37 | (0.20–0.69) |  | 0.37 | (0.19–0.69) |  |
| **Adjuvant regimen** |  |  |  | <0.001 |  |  | <0.001 |
| No adjuvant treatment | 26/84 | 4.91 | (2.70–8.91) |  | 4.58 | (2.51–8.39) |  |
| FOLFOX6 | 19/215 | 1 |  |  | 1 |  |  |
| Capecitabine | 4/33 | 1.53 | (0.52–4.49) |  | 1.46 | (0.49–4.32) |  |
| **WHO performance status** *– (MD=5)* |  |  |  | 0.59 |  |  |  |
| 0 | 40/259 | 1 |  |  |  |  |  |
| 1 | 9/68 | 0.82 | (0.40–1.69) |  |  |  |  |
| **Tumor site (distance to anal verge)** |  |  |  | 0.07 |  |  | 0.07 |
| Low (≤5 cm) | 25/118 | 1 |  |  | 1 |  |  |
| Medium (5.1–10 cm) | 22/171 | 0.63 | (0.35–1.11) |  | 0.67 | (0.37–1.19) |  |
| High (10.1–15 cm) | 2/43 | 0.24 | (0.06–0.99) |  | 0.21 | (0.05–0.91) |  |
| **Extramural spread of the tumor to the perirectal fat** |  |  |  | 0.11 |  |  | ^2^ |
| <5 mm | 17/145 | 1 |  |  |  |  |  |
| ≥5 mm | 32/187 | 1.61 | (0.89–2.90) |  |  |  |  |
| **Initial TNM stage** |  |  |  | 0.36 |  |  |  |
| I/II | 3/31 | 1 |  |  |  |  |  |
| III/IV | 46/301 | 1.73 | (0.54–5.56) |  |  |  |  |

*Abbreviations: HR, hazard ratio; 95% CI, 95% confidence interval; MD, number of patients with missing data;* *PPI, proton pump inhibitor; WHO: World Health Organization*

*^1^The HRs were estimated in the multivariable model and included the following variables:* *PPI exposure, sex, randomization group, adjuvant regimen, and tumor site (distance to anal verge).*

*^2^This variable was not included in the multivariable model because it was correlated to the tumor site (p=0.04).*

**Supplementary Figures**


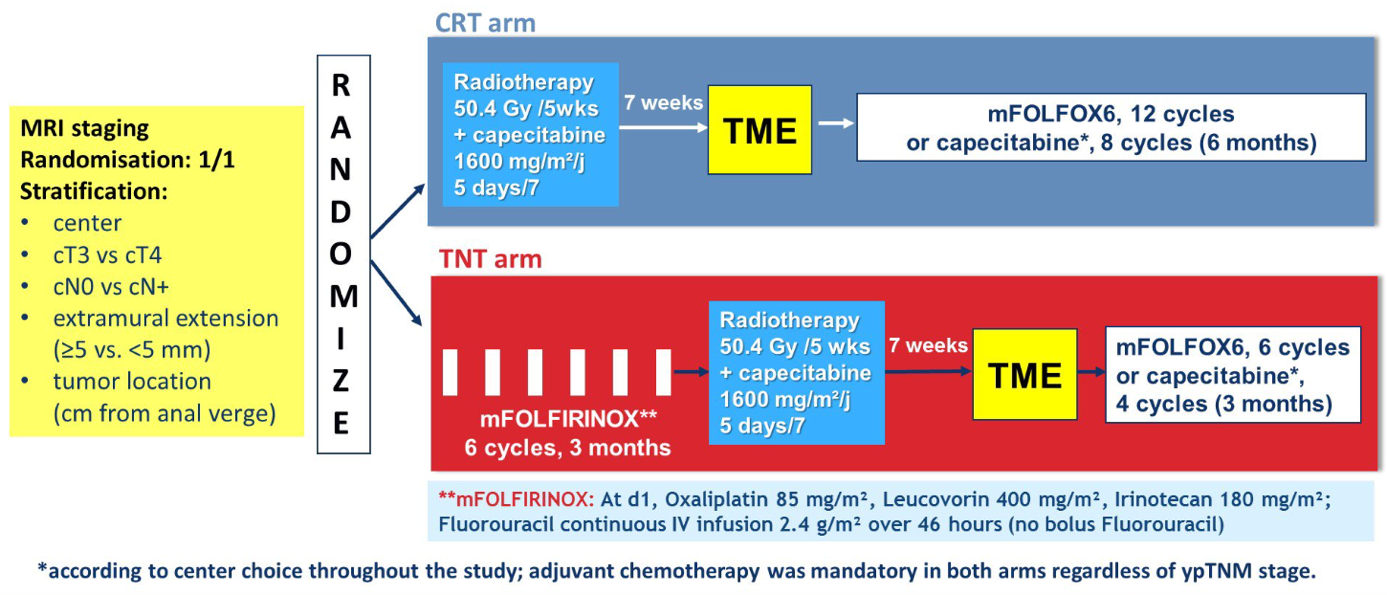


Supplementary Figure 1. Overall scheme of the PRODIGE 23 randomized trial.

Supplementary Figure 2. Kaplan–Meier estimate of the recurrence-free survival curve in the patients selected for the current analysis versus those who were not, but within the PRODIGE 23 trial population.
